# Cryo-EM at ACA 2022

**DOI:** 10.1107/S2052252522009721

**Published:** 2022-10-14

**Authors:** Sriram Subramaniam, Abhay Kotecha, Joseph H. Davis

**Affiliations:** a Department of Biochemistry and Molecular Biology, University of British Columbia, Vancouver, BC, Canada; b Gandeeva Therapeutics Inc., Vancouver, BC, Canada; c Materials and Structural Analysis Division, Thermo Fisher Scientific, Eindhoven, The Netherlands; d Computational and Systems Biology Graduate Program, Department of Biology, Massachusetts Institute of Technology, Cambridge, MA, USA

**Keywords:** cryo-EM, ACA 2022, editorial

## Abstract

The 2022 meeting of the American Crystallographic Association in Portland was an inspiring event, addressing a range of both conventional and emerging themes in structural biology. The increasing emphasis at the conference on methods outside the conventional envelope of crystallography, especially cryo-electron microscopy, is discussed.

The American Crystallographic Association (ACA) was formed in 1949. For over seven decades, it has been the home of all things related to crystallography and the frontier society advancing the study of the arrangement of atoms in matter. In 2021, the ACA rebranded itself as the ‘The Structural Science Society’ to acknowledge the growing emergence of a broader set of methods that inform atomic scale structure. This evolution was very much in evidence at the 2022 Annual meeting of the ACA in Portland, Oregon, particularly so with regard to cryo-electron microscopy (cryo-EM). Numerous talks and sessions at the meeting featured insights obtained from cryo-EM, providing demonstrable evidence that the ACA indeed seeks to reflect the growing importance of non-crystallographic methods in contributing to our understanding of structures at the atomic level in chemistry, materials science and biology.

The adoption of cryo-EM by crystallographers has been fueled by the rapid growth in the number of institutions that are now routinely employing these methods for protein structure determination at high resolution and Fig. 1 ▸ illustrates cryo-EM’s growing impact. Methods closely related to cryo-EM have also seen significant growth, notably cryo-electron tomography (cryo-ET) and micro-electron diffraction (microED). Cryo-ET offers a unique approach of imaging biological complexes in near-native states, and, in combination with methods for sub-tomogram averaging, can generate molecular structures at resolutions approaching those obtained by cryo-EM of purified protein complexes. MicroED enables determination of structures from protein as well as small molecule crystals which are otherwise not accessible by X-ray crystallography because of the small size of the crystals. Below, we highlight selected examples of some of the topics discussed at the meeting that are likely to be of broad and general interest.

The use of cryo-EM for drug discovery has historically been challenging because of limitations in the resolution and size of proteins that could be imaged. However, recent technological advances, including the development of direct electron detectors, energy filters, more effective and automated data collection workflows, and computational data analysis techniques have revolutionized the utility of cryo-EM for visualizing protein-bound small molecule ligands in much the same way that has been possible with X-ray crystallography. Basil Greber and colleagues from the Institute of Cancer Research, London, presented an impressive structural characterization of the 85 kDa CDK-cyclin module of the human CDK-activating kinase bound to about 20 small molecule inhibitors at resolutions in the range of ~2 Å. In another important application that transcends crystallography, Chi-Min Ho and colleagues from Columbia University presented ongoing efforts to study host–pathogen interactions of malarial parasites and to define the structural mechanisms by which *P. faciplarum* hijacks human red blood cells. To obtain large quantities of properly folded proteins the team engineered affinity-tagged parasites using CRISPR-Cas9 technology, cultured them in human erythrocytes and purified the PTEX complex that enabled high-resolution cryo-EM structure determination. Cryo-ET of thin lamella prepared by focused ion-beam milling provided further insights into the *in situ* organization of these complexes.

The emergence of SARS-CoV-2 and the ongoing COVID19 pandemic have resulted in an intense focus on elucidating the fundamental mechanisms that enabled SARS-CoV-2 to spread across the globe and on discovering therapies and vaccines to combat the virus. The spike protein decorates the surface of SARS-CoV-2, is required for infection, and can bind the human protein ACE2, enabling it to enter human cells. Soon after the release of the first genomic sequences of the emerging virus, cryo-EM structures of the SARS-CoV-2 spike protein were reported, and the protein has been a focus of intense scientific scrutiny ever since, and is particularly relevant to understanding the structural landscape of mutations and antibody binding as highlighted by David Veesler from the University of Washington in his presentation.

Crystallographers have almost always had on their hands plenty of microcrystals and nanocrystals produced during crystallization assays are generally too small for collection of complete X-ray diffraction datasets at synchrotron sources. For some challenging crystallization targets, these small crystals are all that can be obtained. This is where microED can help, by taking advantage of the strong interaction that electrons have with matter to extract atomic resolution details of biological material and small molecules from very small crystals. Crystals that are a billionth the size needed for X-ray experiments are exposed to an electron beam in diffraction mode and the data are collected using a fast camera as the sample is continuously rotated in the electron microscope. Atomic resolution structures can be rapidly determined with this approach from a single nanocrystal using the same general instrumental infrastructure that is used for cryo-EM and cryo-ET. Over a dozen presentations at the meeting described the use of microED for solving structures of membrane proteins, small molecules and metal–organic frameworks.

The impact of machine learning in structural sciences featured prominently at the meeting, with AlphaFold and RoseTTAFold’s high-quality structure predictions profoundly influencing how researchers generate, test and refine structure-based hypotheses. The meeting also highlighted the development and application of new machine-learning-based tools throughout cryo-EM and cryo-ET workflows, including hardware and software advances to improve sample preparation, the efficacy of data collection, particle picking, 3D reconstruction, analysis of structural heterogeneity, and inference of conformational free-energy landscapes. One of the compelling takeaways from the meeting was that problems in structural biology that were almost exclusively in the realm of X-ray crystallography are now best addressed by a combination of crystallographic, cryo-EM, cryo-ET, microED and AI/machine-learning-based approaches. Last but not least, Art Olson’s impressive display of the power of bringing advances in virtual reality for collaborative visualization of molecular structures is a reminder of the need to make structural insights easily accessible to all audiences. The future of structural biology now lies in the ability of the community to integrate this broad spectrum of experimental and computational tools in what is certain to be a new and unprecedented era in the description of biology at atomic resolution. Articles describing such developments will continue to be welcomed by **IUCrJ** .

## Figures and Tables

**Figure 1:**
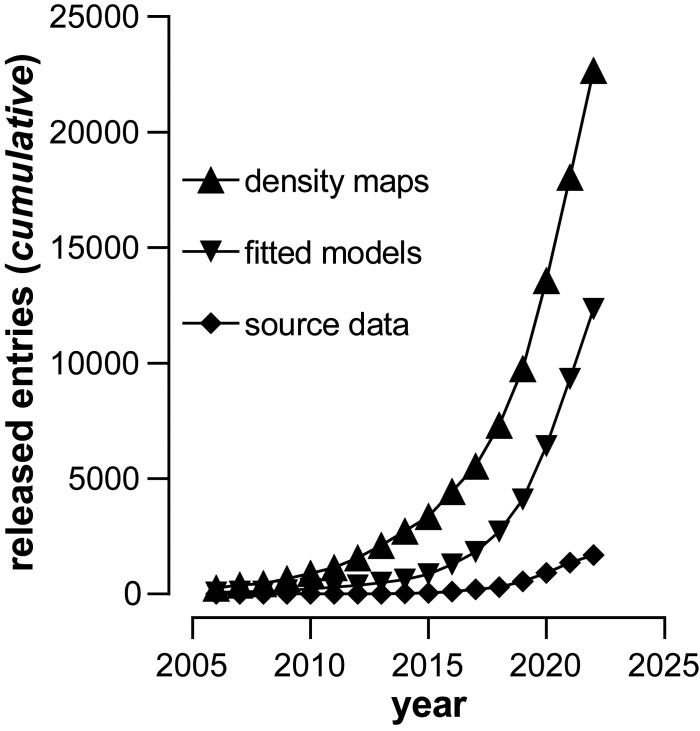
The growing impact of cryo-EM on structural biology. Cumulative number of density maps released at the electron microscopy data bank (triangles), fitted models derived from cryo-EM datasets deposited at the protein data bank (inverted triangles), and source micrographs or particle stacks available at the electron microscopy public image archive (diamonds). Source data were retrieved from the electron microscopy data bank on 6 October 2022.

